# An Unusual Case of Antiphospholipid Syndrome Presenting as Chorea

**DOI:** 10.7759/cureus.3508

**Published:** 2018-10-29

**Authors:** Anudeep Yelam, Pradeep C Bollu

**Affiliations:** 1 Neurology, University of Missouri, Columbia, USA

**Keywords:** antiphospholipid syndrome, chorea, movement disorders

## Abstract

Movement disorders are rarely reported in association with antiphospholipid syndrome (APS). Although it is a rare manifestation in APS, chorea is the most common movement disorder. We report a case of APS in a patient who presented with hyperkinesia, tics, choreiform movements, and other dyskinetic movements involving the mouth and tongue along with behavioral changes. These abnormal movements improved with aripiprazole.

## Introduction

Chorea is defined as involuntary movements that are abrupt, unpredictable, and nonrhythmic, resulting from a continuous random flow of muscle contractions [1]. It is a rare manifestation in antiphospholipid syndrome (APS) and is approximately seen in 1%-2% of patients with this condition. Here, we report a patient with APS who presented with chorea. We will also briefly review the neurological manifestations of APS, the mechanism of chorea in APS, and its management.

## Case presentation

A 60-year-old, right-handed male presented to the emergency room (ER) for acute behavioral changes and abnormal movements for two weeks. He is a truck driver with a remote history of encephalitis and stroke with no baseline deficits. On examination, the patient had stereotypic, “tic-like,” bilateral facial twitches more prominent on the left side, constant winking of the left eye, grimacing facial expressions, seeming to indicate pain along with some tongue thrusting (like a gecko) movements. A video shot by his wife documented these movements. In addition, the patient also exhibited pressured speech and tangential thoughts. On the Montreal Cognitive Assessment (MoCA) scale, the patient scored 20/30, scoring poorly on language, word recall, and calculation.

**Figure 1:**
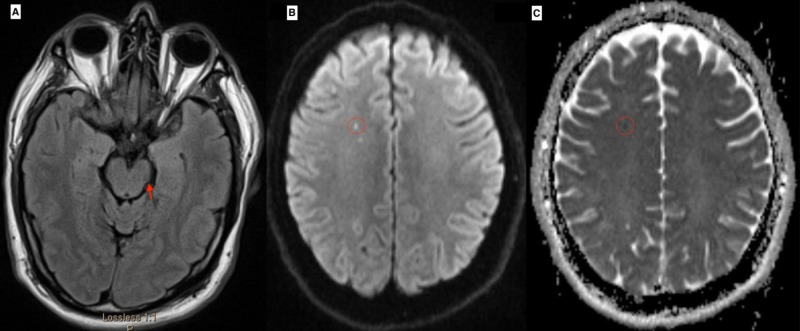
Initial magnetic resonance imaging (MRI) of the brain on axial sequence at the level of the midbrain shows a lacune (arrow) on T2 fluid attenuated inversion recovery (FLAIR), suggestive of an old infarct (1A). On follow-up one month later, a tiny punctuate infarct at the level of the centrum semiovale in the right frontal region (1B) (circle) with the corresponding apparent diffusion coefficient (ADC) correlate (1C) (circle) is seen.

Given his acute behavioral changes and abnormal movements, a computed tomography (CT) scan of the head, an electroencephalogram (EEG), a lumbar puncture with cerebrospinal fluid (CSF) analysis, complete metabolic profile (CMP), complete blood counts (CBC), urine drug screen, serum, and CSF paraneoplastic panel, iron/ferritin levels, and serum copper were ordered to rule out limbic encephalitis, seizures, and infection. All the tests were unremarkable. Magnetic resonance imagining (MRI) of the brain on axial sequence showed a lacune at the level of the midbrain on T2 fluid attenuated inversion recovery (FLAIR) suggestive of an old infarct (Figure 1A).

He was seen five days later in the outpatient neurology clinic and was noted to be much calmer and interacted well. He was started on ziprasidone by his primary care physician for suspected schizophrenia, which helped with the behavioral changes and abnormal movements. Upon further questioning, it was noted that the patient had behavioral changes many months prior to the acute onset of abnormal movements.

On his second follow-up visit one month later, it was noted that he was started on clopidogrel for deep vein thrombosis (DVT) of the right calf that happened in the interim. On examination, the patient had dyskinetic movements involving the mouth and the tongue. Choreiform and occasional high-amplitude ballistic movements are noted in the left upper and lower extremities.

Further workup, consisting of a paraneoplastic panel, vasculitis panel, myasthenia panel, CSF oligoclonal bands, comprehensive drug screen, and CT of chest, abdomen, and pelvis, revealed no abnormalities. CT angiogram of the head did not reveal irregular narrowing or dilatation. MRI of the brain revealed a tiny stroke at the level of the centrum semiovale in the right frontal region (Figures 1B-1C). A coagulation panel showed an elevated phospholipid IgM antibody and anti-beta 2 glycoprotein 1 (B2GP1) antibody.

The diagnosis of APS presenting with chorea was made, and the patient was started on aripiprazole, which improved the symptoms.

## Discussion

The diagnosis of antiphospholipid syndrome (APS) is made in this patient based on the clinical findings, history of deep vein thrombosis (DVT), imaging, elevated phospholipid IgM antibodies, and anti-beta2-glycoprotein 1 (B2GP1) antibodies.

**Figure 2:**
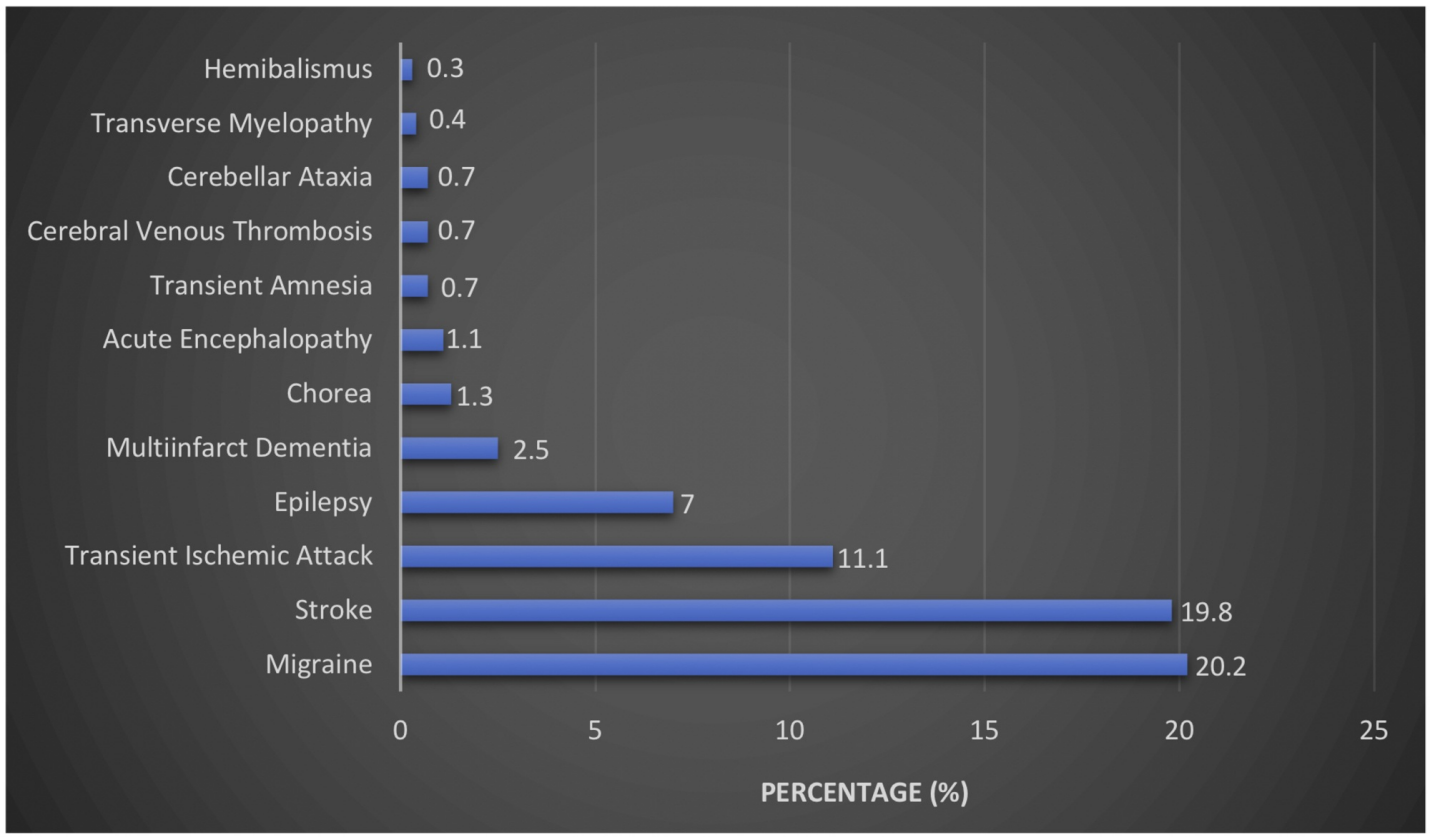
Chart demonstrating the most frequent neurologic manifestations during the evolution of antiphospholipid syndrome (APS).

Hughes, in his original description [2], predicted the importance of neurological manifestations in patients with APS. APS can present with numerous neurological manifestations (Figure 2), among which migraine, stroke, and transient ischemic attack are the commonest [3].

Chorea is a very rare manifestation in APS and can involve any part of the body, including the head and extremities. It can be unilateral or bilateral. Cervera et al. [4] reviewed the clinical, radiological, and immunological characteristics of 50 patients with chorea and APS. Of these, 30% had primary APS and 66% had a single episode of chorea. The mean age of the patients in this study is 21 +/- 12 years (range 6-77 years) with an onset of chorea occurring at 60 years or later in 4%. CT and MRI reported cerebral infarction in 35% of these patients.

The mechanism of chorea in APS is unclear. It is thought that the antiphospholipid antibody (aPL) results in central nervous system (CNS) damage by binding to the endothelium of the brain vessels and causes endothelial dysfunction. This, in turn, increases the vascular permeability and breakdown of the blood-brain barrier (BBB), thus contributing to a hypercoagulable state and thrombus formation [5-7]. The mechanism by which aPL causes chorea is by an antigen/antibody complex binding to the phospholipids in the basal ganglia [8]. Furie et al. and his team demonstrated the presence of striatal hypermetabolism in a young woman with alternating hemichorea and primary APS [9]. During the episodes of hemichorea, the striatal glucose metabolism was noted to be elevated predominantly on the side, contralateral to the abnormal movements. Similarly, during the asymptomatic period, caudate metabolism was also significantly increased contralateral to the body side that later became symptomatic. This suggests that striatal metabolism is a functional concomitant of chorea.

There is currently no diagnostic criteria for aPL-related chorea. The presence of choreiform movements in patients with APS is suggestive of this condition. A careful clinical assessment, exclusion of other neurological conditions, combined with laboratory evaluation and neuroradiologic exams may exclude the other causes of chorea. APS diagnostic criteria [10] do not consider neurologic manifestations, although there is emerging evidence that, neurological disorders may not be uncommon in the setting of APS. The diagnosis of APS is based on a combination of clinical features and laboratory findings like anticardiolipin antibodies (aCL), anti beta2-glycoprotein 1 (B2GP1) antibodies, and Lupus anticoagulant (LA). Although the Sapporo classification criteria are designed for research purposes, they can be used as a reference for the diagnosis of APS [10].

Corticosteroids, anticoagulants, aspirin, and dopamine receptor antagonists have been reported to be effective in patients with APS chorea [4,11]. Neuroleptics such as haloperidol can be tried to control the hyperkinetic movements. Atypical neuroleptics can also be used as alternatives. Our patient improved on aripiprazole. In the absence of remission, the combination therapy, including neuroleptics and steroids, can be tried [4]. Anticoagulation should be reserved for thrombosis treatment and not simply for chorea in the presence of aPL, as it could result in infarction, bleeding, and death [12]. As APS in an autoimmune disorder, the use of immunomodulatory agents has been proposed [13]. But, there is a lack of enough evidence for the selection of specific immunomodulators. Hydroxychloroquine and statins have been tried in patients with recurrent thrombosis despite adequate anticoagulation [14-15]. Rituximab, an anti-CD20 monoclonal antibody can be used in aPL positive patients with hematological manifestations [16]. Anticoagulants, systemic glucocorticoids, plasma exchange, and intravenous immune globulin (IVIg) are generally reserved for patients with catastrophic APS [17]. Immunosuppression with cyclophosphamide or azathioprine has been reported in a very few cases of APS chorea [18-19].

Chorea in aPL is a risk marker for valvulopathy, arterial thrombotic events, and obstetric morbidity [20]. So, the early diagnosis and management of aPL-associated chorea are important to prevent these complications.

## Conclusions

APS can present with a wide range of neurological manifestations. Chorea in the setting of APS is rare, and it can be an initial presentation of APS. It is important to consider APS in the differential and intervene early in the disease process to minimize the complications.
